# Disguised Threat: Rhino-Orbital Mucormycosis Masquerading as Acute Dacryocystitis

**DOI:** 10.22336/rjo.2025.23

**Published:** 2025

**Authors:** Sonali Vinay Kumar, Vikas Sharma, Manoj Gopal Madakshira, Vinay Kumar, Natasha Vinay Kumar, Alok Sati

**Affiliations:** 1 Ophthalmology Department, Command Hospital Eastern Command, Kolkata, India; 2 ENT Department, Command Hospital Eastern Command, Kolkata, India; 3 Pathology Department, Command Hospital Eastern Command, Kolkata, India; 4 Anatomy Department, JIS School of Medical Science and Research, Howrah, India; 5 Department of Medicine, Sri Devaraj Urs Medical College, Kolar, India; 6 Ophthalmology Department, 471 Field Hospital, Arunachal Pradesh, India

**Keywords:** diagnosis, threat, mucormycosis, dacryocystitis, morbidity, ROPLAS = Regurgitation on pressing lacrimal sac, CT = Computed tomography

## Abstract

Mucormycosis is an aggressive, life-threatening fungal infection predominantly affecting immunocompromised individuals. It typically manifests as rhino-orbital or rhino-cerebral disease. However, rarely, its initial presentation may closely resemble an innocuous condition like acute dacryocystitis, which is prone to result in higher morbidity rates. Herein, we report a case of a 52-year-old diabetic patient who presented with symptoms suggestive of acute dacryocystitis. Broad-spectrum antibiotics were started, but the patient did not improve with therapy. Further investigations, including imaging and biopsy, unraveled invasive mucormycosis. Prompt surgical debridement and antifungal therapy were initiated, but the delayed diagnosis culminated in significant orbital involvement. A high index of clinical suspicion is warranted for mucormycosis in patients with risk factors such as diabetes or immunosuppression who present with dacryocystitis and are unresponsive to standard treatment. Early diagnosis and aggressive intervention play a pivotal role in improving patient outcomes in this condition.

## Introduction

Mucormycosis, also called Zygomycosis, is an opportunistic fungal infection caused by mucorales, usually observed among immunocompromised patients [1]. This infection usually begins from the sinonasal mucosa following inhalation of fungal spores, which can later involve the orbit and brain [2]. The sites mainly affected by this condition are the nasal cavity, orbit, central nervous system, lungs, and skin, and sometimes, it can present as a disseminated infection [3]. The most common presentation of this entity is rhino-orbital-cerebral mucormycosis [4]. It usually impinges upon people who are metabolically or immunologically compromised, like diabetic patients, those who are on steroids and chemotherapy, and patients infected with coronavirus disease 2019 (COVID-19) [5]. Mucormycosis is a potentially fatal condition with a tremendous mortality rate when there is extra-nasal involvement. The slight delay in accurately diagnosing this condition can wreak havoc on the patients. The misdiagnosis is very high in the early stage due to a lack of specific clinical features. Rhino-orbital mucormycosis presents with a wide range of symptoms and signs depending upon the extent and location of the infection [6]. Very few documented studies have reported that this disease’s initial presentation can mimic features of acute dacryocystitis. Herein, we report a unique case of mucormycosis in which the patient presented with classic signs of acute dacryocystitis, which delayed appropriate diagnosis and treatment.

Despite initial antibiotic treatment, the condition deteriorated, and further investigation revealed rhino-orbital mucormycosis, which necessitated immediate surgical debridement and antifungal therapy.

## Case presentation

**Fig. 1:**
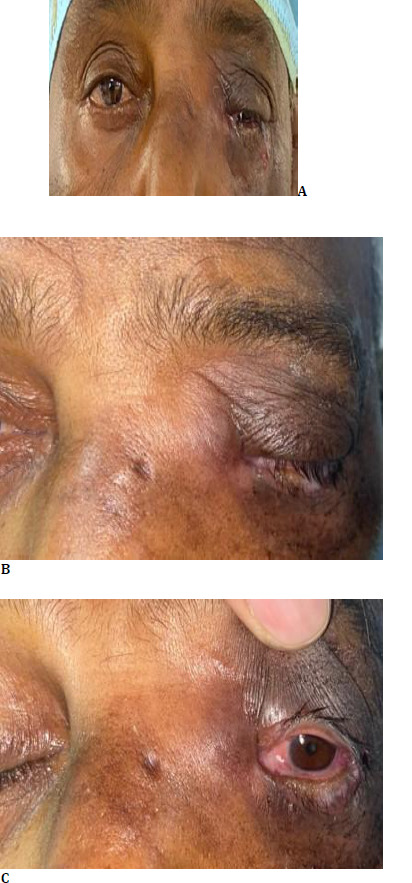
**A-C**. Clinical photograph demonstrating lesion at the inferomedial aspect of the eye and hyperemic conjunctiva

A 52-year-old patient presented at our tertiary care center with complaints of watering from the left eye along with pain, swelling, and redness near the medial canthal area for 2 months. The patient sought consultation for similar complaints at the time of presentation, and he was started on broad-spectrum antibiotics as he was diagnosed with a case of left eye acute dacryocystitis. However, the patient’s symptoms worsened with increased pain and swelling, despite the antibiotic therapy. This compelled him to take a second opinion, and he reported the same complaints at a tertiary care center. The patient also complained of sudden vision loss in the same eye at our center. The patient did not give a history of ocular trauma or prior ocular intervention. There was no history of fever/weight loss/loss of appetite. Clinical examination highlighted that the best-corrected vision in the right eye was 6/9, and hand movement close to the face in the left eye. Pupillary reaction showed relative afferent pupillary defect in the left eye. Ocular movements were full and free in both eyes. Fundus examination showed no abnormality in the right eye and a pale disc in the left eye. A lacrimal irrigation test was done, which revealed patent nasolacrimal duct in the right eye, and in the left eye, regurgitation on pressing the lacrimal sac (ROPLAS positive). Slit lamp examination showed high tear meniscus height in left eye and grade 2 nuclear sclerosis in lens in the both eyes. On clinical examination, a mass was observed in the medial canthal area, which was firm and tender. Posterior extent was not felt, and skin over the lesion was found erythematous (**Fig. 1 A-C**).

**Fig. 2:**
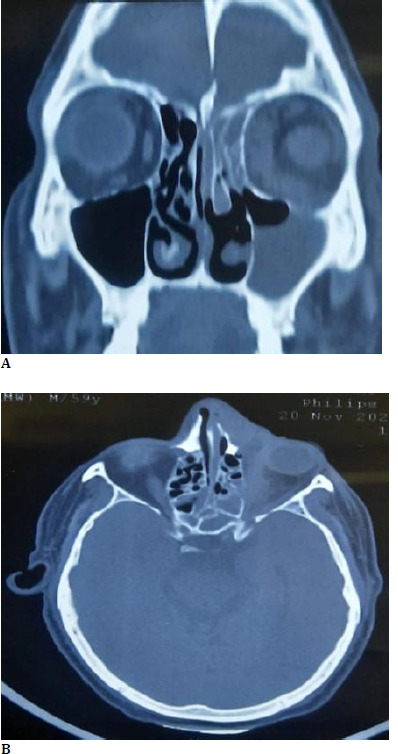
**A, B**. CT orbit and PNS image showing soft tissue content in the left maxillary, ethmoid, and frontal sinus with left intraorbital extension, which is causing erosion of the nasal septum, the cribriform plate, and wall of left maxillary sinus in the coronal and axial can

Computed tomography (CT) orbit was advised as the patient’s condition did not improve despite being started on IV antibiotics. CT orbit showed soft tissue content in the left maxillary, left frontal, bilateral anterior and posterior ethmoidal, and bilateral sphenoid sinus. This soft tissue content extended into the left inferior and medial orbital space through the eroded floor of the orbit and lamina papyracea. Anteriorly, it extended into the nasal cavity and caused erosion of the nasal septum. Superomedially, it was causing erosion of the cribriform plate. Posteriorly, erosion of the wall of the left maxillary sinus with extension into the pterygopalatine fossa and anterior aspect of the infra temporal fossa was observed (**Fig. 2 A, B**).

**Fig. 3:**
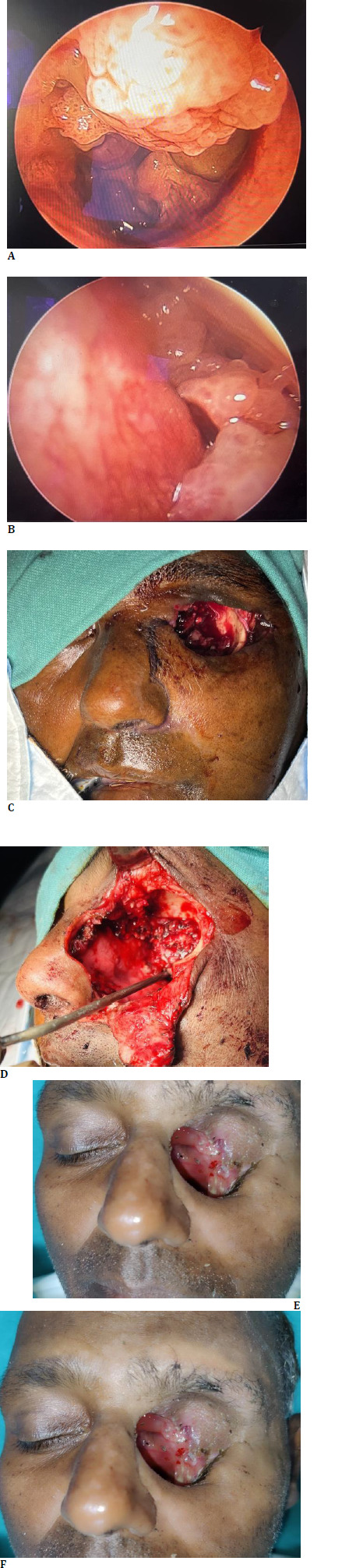
**A, B**. Pre-operative figure showing polypoidal lesion in left nasal cavity filling entire maxillary and ethmoid sinus with extension to roof of nasal cavity; **C, D**. Intraoperative figure showing steps of left orbital exenteration and suprastructure maxillectomy; **E, F**. Post-operative figure showing a well-healed orbital and maxillary cavity

Endoscopic evaluation at our center showed a polypoidal lesion in the left nasal cavity and black necrotic areas over the turbinate (**Fig. 3 A, B**). A biopsy was taken from this site, and histopathological examination showed broad, non-septate hyphae suggestive of mucormycosis.

Upon diagnosis of mucormycosis, endoscopic trans-nasal debridement of ethmoid and maxillary sinus with left maxillectomy and left orbital exenteration were done under general anesthesia (**Fig. 3 C, D**).

**Fig. 4:**
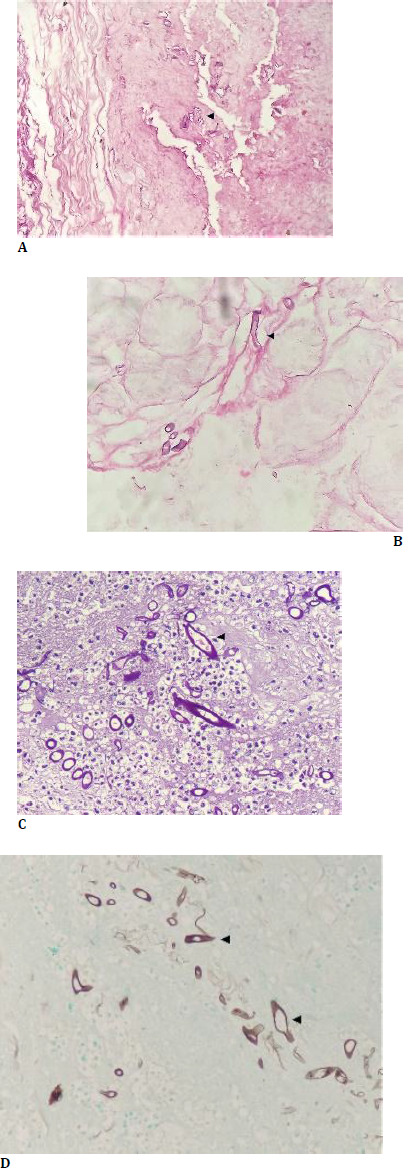
**A**. Hematoxylin and Eosin stain (40x magnification) showing bland necrosis with scattered fungal profiles in the medial part of the orbital soft tissue and eyeball (arrowhead); **B**. H and E stain (100x magnification) showing broad-based and fragmented fungal profiles (arrowhead); **C**. Periodic acid Schiff’s stain (400x magnification) highlights the magenta colored walls of the fungal profiles, which are pauciseptate (arrowhead); **D**. Grocott stain (400x magnification) highlights the brownish black colored wall of the fungal profiles, which are broad, pauciseptate, and show obtuse angle branching pattern (arrowhead)

The patient was started on intravenous antifungal therapy with liposomal amphotericin B (5 mg/kg body wt.) for 6 weeks, which is the standard treatment for mucormycosis. Subsequently, the patient was started on Tab Posaconazole 300 mg HS for 6 months. The patient’s diabetes management was also optimized to improve immune function and overall recovery. The specimen was sent for histopathological examination, confirming the mucormycosis diagnosis (**Fig. 4 A-D**). The patient was followed up for 6 months, and no sign of recurrence was found (**Fig. 3 E, F**).

## Discussion

Mucormycosis is an uncommon, aggressive, fulminating fungal infection that can be fatal if untreated [7]. Due to the fungal hyphae’s vasculature invasion, infarction and tissue necrosis are defining characteristics of mucormycosis [7,8].

One of the lesser-known and rare presentations of mucormycosis is its ability to mimic acute dacryocystitis, which can lead to misdiagnosis and delayed treatment.

Acute dacryocystitis is typically an infection of the lacrimal sac caused by an obstruction of the nasolacrimal duct. Patients often present with pain, swelling, and redness in the region of the lacrimal sac, along with purulent discharge.

Rhino-orbital mucormycosis begins with infection in the nasal or sinus cavities and can rapidly extend into the orbit, brain, and surrounding tissues due to its angioinvasive nature. However, in rare instances, mucormycosis can initially present with symptoms that resemble acute dacryocystitis, misleading the practitioner and delaying the definitive diagnosis.

Reviewing the medical literature, we found that very few studies reported that the initial presentation of mucormycosis can mimic features of acute dacryocystitis.

Kapoor et al. reported mucormycosis infection of the lacrimal sac in an immunocompetent woman who initially presented with epiphora secondary to nasolacrimal duct obstruction. A routine lacrimal sac biopsy was done during dacryocystorhinostomy, revealing mucormycosis [9].

Halawa et al. mentioned lacrimal sac infections caused by mucorales in a diabetic patient and found, in their study, that the patient had no evidence of concurrent sinus involvement [10].

In our case, the patient was initially diagnosed with acute dacryocystitis, a misleading diagnosis that resulted in the deterioration of the condition. The overlapping symptoms of acute dacryocystitis and mucormycosis often lead to an erroneous diagnosis, which happened in our case. The delay in diagnosing mucormycosis due to ambiguous clinical features caused orbital and intracranial spread in the present case.

Dacryocystitis is caused by bacterial infections like staphylococcus aureus or streptococcus, which usually respond to systemic antibiotics, whereas in mucormycosis, antibacterial therapy will not halt the disease progression. The lack of improvement and rapid worsening of symptoms following standard treatment of dacryocystitis should raise suspicion for other possible diagnoses, particularly in immunocompromised patients or those with poorly controlled diabetes. Imaging, such as CT orbit and PNS, played a handy role in diagnosing this condition in the present case as it divulged the invasive nature of the infection and involvement of the sinuses, orbit, and brain. A biopsy of the affected tissue, with fungal culture and histopathological examination, confirmed the diagnosis of mucormycosis in our case. We speculated in our case that fungal spores, after inhalation, might have reached the nasolacrimal duct directly after breaching the nasal mucosa and induced nasolacrimal duct obstruction by generating inflammatory exudate.

This case aimed to draw attention to rhino-orbital mucormycosis's often cryptic and nonspecific initial presentation. A high degree of clinical suspicion is required to diagnose this condition, especially in immunocompromised patients.

## Conclusion

This case underscores the importance of contemplating the diagnosis of mucormycosis in patients presenting with features of acute dacryocystitis who do not respond to standard antibacterial treatment, particularly in those with underlying risk factors like diabetes mellitus. Clinicians should be aware that this potentially fatal infection can masquerade as a more common condition, often leading to delayed treatment and higher morbidity rates.
